# Hyper-Fractionated Radiotherapy for Soft Tissue Sarcoma: Results of the Second Study of Hyper-Fractionated Radiotherapy

**DOI:** 10.1080/13577149977587

**Published:** 1999-12

**Authors:** R. Jacob, D. Gilligan, M. Robinson, C. Harmer

**Affiliations:** 1 Sarcoma unit Royal Marsden Hospital NHS Trust Fulham Road London UK; 2 Department of Clinical Oncology Addenbrooke's Hospital Cambridge UK; 3 Department of Clinical Oncology Weston Park Hospital NHS Trust Sheffield UK; 4 Department of Radiotherapy Velindre NHS Trust Whitchurch Cardiff CF4 7XL UK

## Abstract

*Purpose and Method.* Hyper-fractionated radiotherapy for treatment of soft tissue sarcomas is designed to deliver a higher total dose of radiation without an increase in late normal tissue damage. In a previous study at the Royal Marsden Hospital, a total dose of 75 Gy using twice daily 1.25 Gy fractions resulted in a higher incidence of late damage than conventional radiotherapy using 2 Gy daily fractions treating to a total of 60 Gy. The current trial therefore used a lower dose per fraction of 1.2 Gy and lower total dose of 72 Gy, with 60 fractions given over a period of 6 weeks.

*Subjects.* A total of 37 patients (22 males and 15 females) with a median age of 56 years (range 19–88 years) were treated.

*Results.* Of eight patients treated pre-operatively, six showed a partial response and in two the tumour was static. The maximum acute toxicities were grade 1 in eight, grade 2 in 14 and grade 3 in 15 patients. Late toxicities of the skin were graded 1 in 10 and grade 2 in nine patients. Five patients complained of pain in the irradiated bone and soft tissues, which was of moderate severity (grade 2). Stiffness was graded 2 in three patients and severe (grade 3) in one.Three patients had moderate and one patient had severe lymphoedema following treatment. The 5-year recurrence-free survival probability of patients treated radically was 76%. Following excision of local recurrences the study group had a disease-free survival probability of 86% at 5 years.

*Discussion.* The regime is well tolerated with comparable local control and late complication rates to standard daily fractionated therapy.The potential benefit of this regime needs to be defined in a prospective randomized trial.


*Sarcoma* (1999) **3**, 157–165

ORIGINAL ARTICLE

### Hyper-fractionated radiotherapy for soft tissue sarcoma: results of the second study of hyper-fractionated radiotherapy

R. JACOB,[1] D. GILLIGAN,[2] M. ROBINSON[3] & C. HARMER[1]

[1]*Sarcoma unit, Royal Marsden Hospital NHS Trust, Fulham Road, London, UK,* [2]*Department of Clinical Oncology, Addenbrooke's Hospital, Cambridge, UK,* [3]*Department of Clinical Oncology, Weston Park Hospital NHS Trust, Sheffield, UK*

**Abstract**

*Purpose and Method.* Hyper-fractionated radiotherapy for treatment of soft tissue sarcomas is designed to deliver a higher total dose of radiation without an increase in late normal tissue damage. In a previous study at the Royal Marsden Hospital, a total dose of 75 Gy using twice daily 1.25 Gy fractions resulted in a higher incidence of late damage than conventional radiotherapy using 2 Gy daily fractions treating to a total of 60 Gy. The current trial therefore used a lower dose per fraction of 1.2 Gy and lower total dose of 72 Gy, with 60 fractions given over a period of 6 weeks.

*Subjects.* A total of 37 patients (22 males and 15 females) with a median age of 56 years (range 19–88 years) were treated.

*Results.* Of eight patients treated pre-operatively, six showed a partial response and in two the tumour was static. The maximum acute toxicities were grade 1 in eight, grade 2 in 14 and grade 3 in 15 patients. Late toxicities of the skin were graded 1 in 10 and grade 2 in nine patients. Five patients complained of pain in the irradiated bone and soft tissues, which was of moderate severity (grade 2). Stiffness was graded 2 in three patients and severe (grade 3) in one. Three patients had moderate and one patient had severe lymphoedema following treatment. The 5-year recurrence-free survival probability of patients treated radically was 76%. Following excision of local recurrences the study group had a disease-free survival probability of 86% at 5 years.

*Discussion.* The regime is well tolerated with comparable local control and late complication rates to standard daily fractionated therapy. The potential benefit of this regime needs to be defined in a prospective randomized trial.

**Key words**: Hyper-fractionation, radiotherapy, sarcomas

### Introduction

Radiotherapy is an essential component of treatment for all soft tissue sarcomas (STS) except those which have been widely excised or are of low grade. Conservative surgery and post-operative radiotherapy is the preferred procedure for limb and limb-girdle sarcomas, with 5-year local control rates of 80–90% reported in different series.[1–4] Pre-operative radiotherapy may be used (with or without chemotherapy) in tumours not initially amenable to limb conserving surgery and around a 30% improvement in the rate of limb conservation has been reported.[5–7]

Despite best efforts at conservative surgery and conventional radiotherapy, most studies report local recurrence rates of up to 20% at 5 years; late local failure can continue to occur beyond that period.[1,2] Tumours of higher grade and larger size are particularly at risk of local failure.[2,8,9] Local recurrence is observed in about one fifth of patients with metastases,[2] although it is uncertain whether the risk of distant metastases is increased following local recurrence.[2,9] Around 60% of local recurrences can be controlled with further surgery but amputation may be necessary.[2,5] Studies aimed at further improving local control of extremity STS are hence of paramount importance.

It appears that conventional radiotherapy has reached the limit of its performance and it is imperative that newer modalities/techniques be tested in an attempt to improve results. Conservative surgery and brachytherapy is reported successfully to control locally advanced sarcomas of the limb with good preservation of function.[10] Better target precision and tumour oxygenation are postulated advantages with this approach but no significant improvement in overall results has been observed with brachytherapy over conventional external beam radiotherapy.[10,11] Improved results are reported with the addition of infusional chemotherapy to radiotherapy in the pre-operative setting, although at the expense of

Correspondence to: R. Jacob, MD, Department of Radiotherapy, Velindre NHS Trust, Whitchurch, Cardiff, CF4 7XL, UK. Fax: 01222 522694; Email: rojymon@hotmail.com

1357-714X print/1369-1643 online/99/040157-09 © 1999 Taylor & Francis Ltd additional toxicity.[12–14] The initial promise of higher relative biological effectiveness (RBE) with neutron therapy in the treatment of these tumours has also been offset by a decreased therapeutic ratio through increased late damage.[15–17]

The possible benefit of radiotherapy dose escalation in the treatment of STS has not been prospectively tested. Most radiotherapy schedules limit the total dose of post-operative radiotherapy to 60–66 Gy in view of the unacceptably high incidence of late complications with doses above this level. It was in this setting that altered radiotherapy fractionation was evaluated in the post-operative treatment of extremity STS.

The technique of hyper-fractionated radiotherapy can be used to deliver a higher total dose of radiation without an increase in late normal tissue damage.[18] This approach is proven to be of benefit in primary tumours arising in the head and neck, bladder, lung and in gliomas[19,20] but there are few reports on its efficacy in the treatment of STS. Trials of combined chemotherapy and hyper-fractionated radiotherapy in paediatric Ewing's and rhabdomyosarcoma have shown promising results[21,22] and radiotherapy using multiple daily fractions was effective in the pre-operative setting in combination with radiosensitizing agents.[23,24] These observations formed the basis of a pilot study of hyper-fractionated radiotherapy in the treatment of extremity STS at the Royal Marsden Hospital.

The first study of hyper-fractionation was designed to determine the feasibility of irradiating large volumes to a dose of 75 Gy using twice daily 1.25 Gy fractions with a minimum interval of 6 hours between fractions.[25] Assuming on $\alpha/\beta$ value of 1.36 for late responding tissues, the expected late effects with hyper-fractionated regime were equal to conventional irradiation using 2 Gy fractions and a total of 60 Gy. However, it appeared that the late damage caused by this dose greater than with conventional irradiation, suggesting higher $\alpha/\beta$ values for late-responding tissues.

In order to reduce the incidence and severity of late complications, we designed the current trial of hyper-fractionated radiotherapy in extremity STS, assuming a $\alpha/\beta$ of 3 for late reaction. By using a lower dose per fraction of 1.2 Gy and lower total dose of 72 Gy, with 60 fractions being given over a period of 6 weeks, it was expected that the late toxicity would be equivalent to conventional fractionation. Assuming an $\alpha/\beta$ of 10 for tumour, there would be a 11% increase in the effective dose for tumour control.

### Materials and methods

This study was conducted on patients with STS of the extremity referred to the Sarcoma Unit of the Royal Marsden Hospital between 1990 and 1995. All patients were initially assessed by the multidisciplinary team comprising surgeon, radiotherapist and medical oncologist. Initial investigations included computed tomography/magnetic resonance imaging (CT/MRI) scans of the primary site and CT scan of the chest. Patients referred with no histological confirmation of diagnosis underwent tru-cut biopsy of tumour. All histology specimens were reviewed by the same pathologist and graded as high, intermediate or low.

Surgical excision of the tumour was the preferred mode of treatment whenever possible and patients who achieved wide excision of a low grade tumour were not offered post-operative radiotherapy. All patients with intermediate or high grade tumours and those in whom tumour was not excised with wide margins, received post-operative radiotherapy. This included patients who had 'intra-capsular' or 'marginal' resection of tumours by the Enneking's classification.[6] Pre-operative radiotherapy was given to patients whose tumours were not initially amenable to limb-conserving surgery. Palliative radiotherapy was given for patients with poor performance status or documented metastatic disease. No patient received chemotherapy as part of their primary treatment.

All patients requiring radiotherapy were considered for treatment using the hyper-fractionated protocol. Presence of bowel or neural tissue in the target volume was the only criterion for ineligibility, as the study design did not consider the $\alpha/\beta$ ratios and sensitivities of these structures which are different to soft-tissue. During this time period, the large majority of patients were treated conventionally. Only those who were able to attend for twice daily radiotherapy could be included in the study, with most being admitted to the ward. Written informed consent was mandatory.

Patients were immobilized using a perspex cast and treatment was planned using a CT scan, for 5/6 MV photons in two phases. Phase I volume included the whole muscle compartment of the limb, usually with opposing fields, angled if necessary, with or without compensators. For phase II a much smaller volume encompassing the initial extent of the primary tumour with a 2 cm margin was used. Care was taken to leave a corridor of normal tissue un-irradiated in all patients and to spare the palm, sole, heel, Achilles tendon, toes and joints whenever possible.[26–28] The principles and techniques of three-dimensional conformal radiotherapy have been described previously.[29]

A phase I dose of 60 Gy followed by a phase II dose of 12 Gy in 1.2 Gy fractions treating twice a day was given in 60 fractions over 6 weeks post-operatively. Alternatively, pre-operative radiotherapy was given to the phase I volume to 60 Gy over 5 weeks following which patients were considered for surgery; tumours which were still not amenable to limb-conserving surgery were treated further using radiotherapy to a phase II volume, to a total dose of 72 Gy. Palliative irradiation was also given to a total dose of 72 Gy in

**Table 1.** *RTOG acute radiation morbidity scoring criteria*

| 0 | 1 | 2 | 3 | 4 |
|---|---|---|---|---|
| No change | Erythema/epilation/dry desquamation | Tender or bright erythema/patchy moist desquamation/ moderate oedema | Confluent moist desquamation/pitting oedema | Ulceration/haemorrhage/ necrosis |

two phases. There was always a minimum gap of 6 h between the two daily fractions.

Patients were monitored weekly during radiotherapy: skin erythema, desquamation, oedema and ulceration were recorded. Toxicity was graded according to the RTOG radiation morbidity scoring criteria (Table 1).

Tumour response was assessed 4 weeks after the end of phase I treatment in patients treated pre-operatively. Tumours with less than 50% reduction in the product of two perpendicular dimensions were assessed as having 'static disease'. A reduction in dimensions of 50% or more but short of complete response was designated a 'partial response'. Patients with 'static disease' were suitable for surgery if the tumour had become technically operable.

Patients were reviewed at 3-monthly intervals during the first 2 years and at longer intervals thereafter, for evaluating disease status and scoring late toxicity. RTOG/EORTC late radiation morbidity scoring scheme was used to study the late effects on skin, sub-cutaneous tissue, bone and joint; the NCIC late limb oedema scale was used to score post-treatment oedema (Table 2). Follow-up CT/MRI scans of the limb and chest were performed whenever clinically indicated.

Recurrence-free survival was calculated using the Kaplan–Meier product limit method. Prognostic variables for radiation reactions were studied using the Chi-squared test.

### Results

### *Patient characteristics (Table 3)*

Thirty-seven patients underwent treatment using the hyperfractionated regime. There were 22 males and 15 females with a median age of 56 years (range 19–88 years). Thigh was the commonest site of presentation, in 15 patients. The other common sites of tumour were lower leg ($n$=10) upper arm, and foot ($n$=3 each). Leiomyosarcoma ($n$=14) and malignant fibrous histiocytoma ($n$=12) were the most frequent histological subtypes; in two patients the type was not specified (NOS). In 10 patients the tumour was of intermediate grade and in 27 patients high grade. Twenty-nine patients were treated post-operatively and eight pre-operatively. Post-operative radiotherapy was given for high grade tumours in 23 patients and for close margins of excision in six patients.

Thirty patients were treated with radical intent: 27 post-operatively and three pre-operatively. Seven patients were treated with palliative intent because of documented metastatic disease but to the same dose. This included five pre-operative and two post-operative radiotherapy.

### *Tumour response and local control (Table 4)*

Of the eight patients treated with pre-operative radiotherapy, six patients showed partial response with more than 50% reduction in tumor size and in two

**Table 2.** *RTOG late radiation morbidity scoring criteria and NCIC late limb oedema scale*

| | 0 | 1 | 2 | 3 | 4 |
|---|---|---|---|---|---|
| Skin | None | Slight atrophy/ pigmentation/some hair loss | Patchy atrophy/moderate telangiectasia/total hair loss | Marked atrophy/gross telangiectasia | Ulceration |
| Subcutaneous tissue | None | Slight induration/Loss of sub-cutaneous fat | Moderate fibrosis/slight field contracture contracture/<10% linear reduction | Severe induration/field contracture/>10% linear measurement | Necrosis |
| Bone | None | Asymptomatic/no growth retardation/reduced bone density | Moderate pain/growth retardation/irregular bone sclerosis | Severe pain/complete arrest of bone growth/dense bone sclerosis | Necrosis/spontaneous fracture |
| Joint | None | Mild joint stiffness/limitation of movement | Moderate stiffness/joint pain/limitation of movement | Severe joint stiffness/pain/severe limitation of movement | Necrosis/complete fixation |
| Oedema | None | Slight but definite | Moderate | Considerable swelling | Skin shiny and tight |

**Table 3.** *Patient characteristics*

| Characteristics | Post-operative | Pre-operative |
|---|---|---|
| Number | 29 | 8 |
| Gender | | |
| Male | 17 | 5 |
| Female | 12 | 3 |
| Mean age (years) | 56 | 56 |
| Mean tumour size (cm) | 9.1 | 9.5 |
| Site | | |
| Thigh | 13 | 2 |
| Lower leg | 6 | 4 |
| Other | 10 | 2 |
| Histology | | |
| LMS[1] | 12 | 2 |
| MFH[2] | 8 | 4 |
| Others | 9 | 2 |
| Grade | | |
| Intermediate | 6 | 4 |
| High | 23 | 4 |

1, Leiomyosarcoma; 2, malignant fibrous histiocytoma.

the tumour was static with less than 50% reduction. All three patients treated with radical intent had partial response and underwent limb-conserving surgery. Surgery was performed after 60 Gy in one patient and after 72 Gy in two patients. All three patients died of distant metastases. One patient also had evidence of local recurrence at 18 months after surgery. Of the five patients treated with palliative intent, three showed partial response to irradiation and the disease was static in the remaining two. One patient with static disease underwent limb amputation. All five patients died of distant metastases.

Twenty-nine patients received post-operative radiotherapy. Two patients who received post-operative radiotherapy with palliative intent died of metastatic disease, with no evidence of local recurrence. Five of 27 patients treated with radical post-operative radiotherapy developed local recurrence of tumour. Three of these patients had excision of recurrence and are alive and free of disease. The other two patients had metastatic disease such that no further surgery was offered. Both these patients died of metastatic disease before the recurrent tumour became symptomatic. Nine patients died of metastatic disease without evidence of local recurrence.

Of 30 patients treated radically (27 with post-operative and three with pre-operative radiotherapy) there were a total of six local failures (five and one, respectively). Sixteen patients developed distant metastases of whom three also failed locally. The mean time to develop metastases was 23 months (range 1–92 months).

All seven patients treated palliatively (two with post-operative and five with pre-operative radiotherapy) died of distant metastases. Four patients treated with pre-operative radiotherapy showed no evidence of tumour progression at the time of death. One patient with ‘static’ response to pre-operative irradiation had subsequent tumour progression of disease and underwent amputation of the limb.

Thirty-five patients completed the planned course of treatment without interruption. Treatment was stopped in two patients treated post-operatively at 58.8 and 64.8 Gy due to break-down of the surgical flap.

Patients in this study had a median follow up of 44 months (range 2–114). The 5-year recurrence-free survival probability in patients treated with radical intent using pre- or post-operative hyperfractionated radiotherapy, was 76%. The 5-year disease-free survival after surgical salvage was 86%.

### *Early reactions*

The maximum acute toxicity was only grade 1 in eight patients and 14 patients had grade 2 toxicity. Grade 3 toxicity was recorded in 15 patients, but none developed ulceration or necrosis (grade 4 toxicity). Nine patients had breakdown of their grafts while on radiotherapy not graded as grade 4 radiation toxicity. Breakdown of surgical scars/grafts could not be entirely attributed to radiotherapy treatment, and graft breakdown occurred before the onset of grade 2 or 3 toxicity in six patients. Graft breakdown was complicated by infection in three patients and, in the other six, the breakdown was minor, healing with conservative management.

**Table 4.** *Hyper-fractionated radiotherapy in STS: treatment outcome*

| Treatment | Intent | Complete response | Partial response | Static | Local failure | Distant failure | Both | Disease-free |
|---|---|---|---|---|---|---|---|---|
| Post-operative ($n$=29) | Radical ($n$=27) | — | — | — | 3* | 9 | 2 | 16* |
| | Palliative ($n$=2) | — | — | — | — | 2 | 0 | 0 |
| Pre-operative ($n$=8) | Radical ($n$=3) | — | 3** | — | — | 2 | 1 | 0 |
| | Palliative ($n$=5) | — | 3 | 2 | — | — | 5 | 0 |

*All three patients underwent excision of local recurrence and are alive disease-free.
**Three patients underwent complete surgery after pre-operative radiotherapy.

Figure 1 shows the time in weeks at which toxicities developed. Acute reactions generally developed during the second week of treatment and more than 60% of grade 1 toxicities occurred by the end of week 3. Grade 2 toxicity peaked around the fifth week of radiotherapy and continued to develop throughout the duration of treatment. Progression to grade 3 toxicity was common around week 6. Acute radiation reactions were the same within the phase I and II radiation fields and there was no increase in incidence or severity of acute toxicity within the phase II volume.

### *Late reactions*

Late radiation morbidity was assessed in 32 patients. Assessment was not possible in three patients as they died of metastatic disease before the development of late changes. One patient underwent amputation following partial response to pre-operative radiotherapy and one patient was lost to follow-up.

Late morbidity was most commonly observed in the skin and sub-cutaneous tissues. Ten patients had only grade 1 morbidity of the skin with pigmentation and nine patients grade 2 morbidity with moderate telangectasia. In the sub-cutaneous tissue, induration was recorded as slight (grade 1) in 11, moderate (grade 2) in nine and severe (grade 3) in one patients.

Five patients complained of pain in the irradiated bone or soft tissues, which was of moderate severity (grade 2). Five patients had mild (grade 1) stiffness of the irradiated joint. Stiffness was graded 2 in three patients and severe (grade 3) in one. Lymphoedema was slight (grade 1) in 13 patients, moderate (grade 2) in three and considerable (grade 3) in one.

Incidence of grade 2 or 3 late radiation morbidity of the sub-cutaneous tissue, bone or joint were higher in patients treated with phase II field areas greater than 250 $cm^2$, although this was not statistically significant (given the small number of events recorded).

Figures 2–4 show an ulcerated STS treated with wide excision and hyperfractionated radiotherapy, with excellent results.

### Discussion

In the previous study of hyper-fractionated radiotherapy at the Royal Marsden Hospital, a total dose of 75 Gy in twice daily 1.25 Gy fractions was tested. Assuming an $\alpha/\beta$ ratio for late damage of 1.36, it was expected that this regime would give equivalent late toxicity compared to 60 Gy in 30 fractions.[26] The late effects from that study were greater than with conventional fractionation and in our current study an 11% increase in the therapeutic ratio was expected for the same late effects, assuming a higher $\alpha/\beta$ ratio of 3 for late-responding tissues.

Comparison of results with that of our previous study shows a slightly reduced incidence in grade 2–4 acute radiation morbidity (43 vs 48%). The new group of patients have a minimum follow-up period of 33 months, with a median of 44 months, enabling scoring of most late radiation reactions. A shorter median follow-up period in the previous study would suggest that the actual incidence of late toxicity could be even higher than reported. The incidence of grade 2 or 3 induration is 27% in this study contrasted with 53% in the previous study. An overall local control probability of 86% at 5 years in this study compares

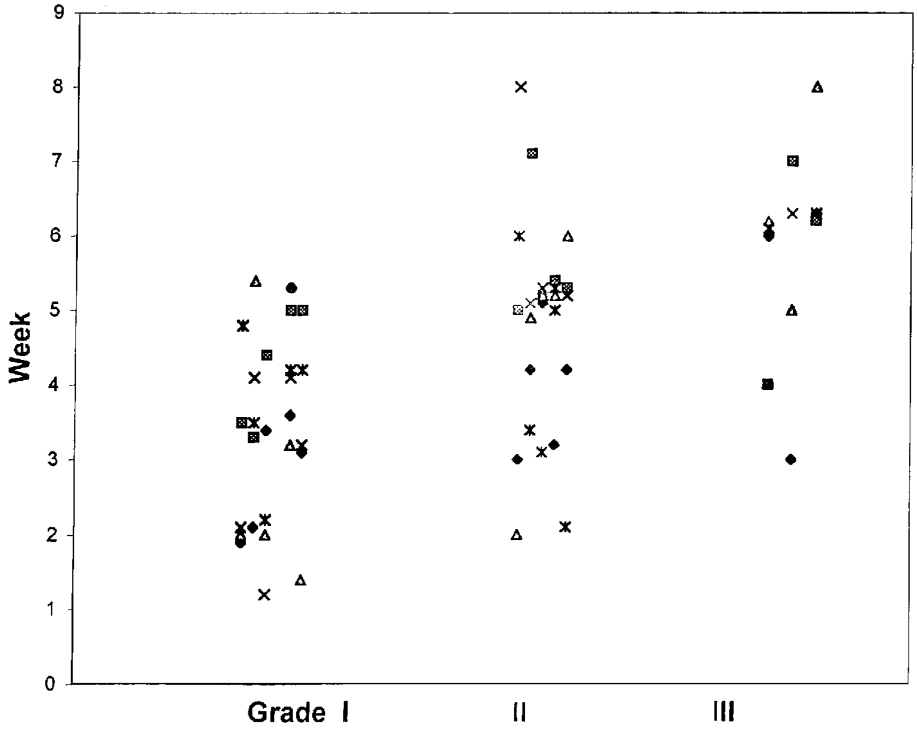

**Figure 1.** *Graph showing the week of onset of acute radiation toxicity.*

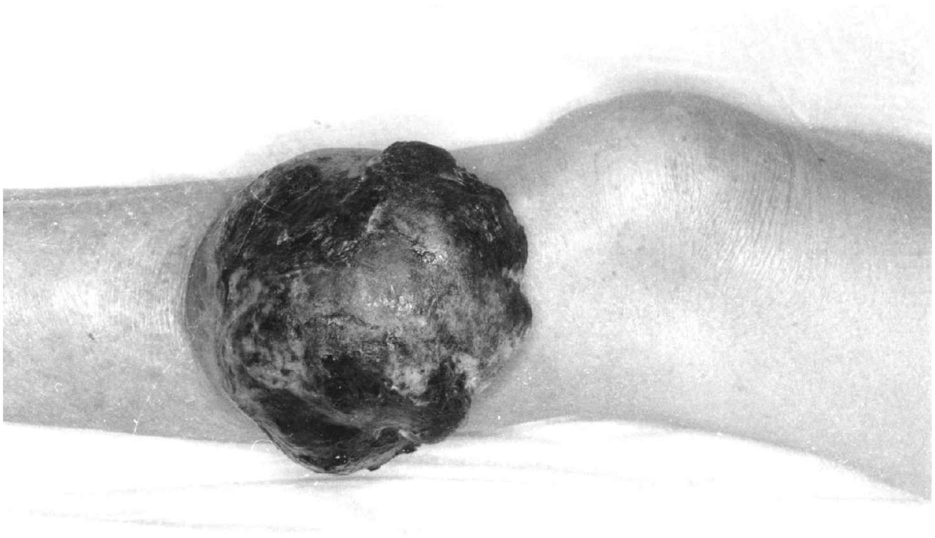

**Figure 2.** *Photograph of an ulcerated soft tissue sarcoma in the upper outer aspect of the lower leg in an 88-year-old woman.*

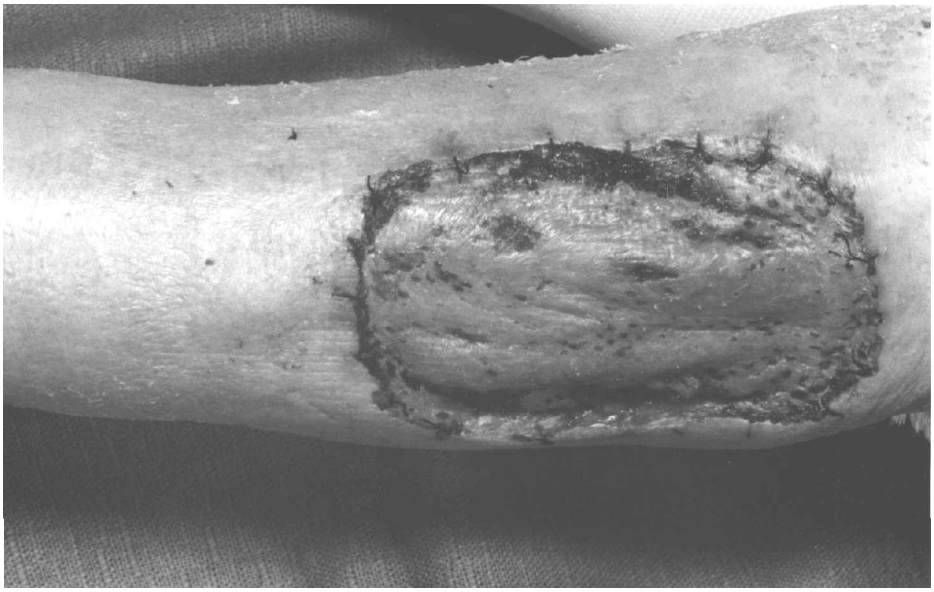

**Figure 3.** *Photograph of the leg in Fig. 2 following wide excision of a 10× 10 cm, grade 3 leiomyosarcoma and repair of the defect.*

favourably with results using conventional radiotherapy.[1,2,30] This suggests that hyper-fractionated radiotherapy using twice daily 1.2 Gy fractions is well tolerated with good local control rates and no increase in the incidence of late toxicity.

There is some evidence to suggest that soft tissue sarcomas show a dose response to irradiation, although this has not been proven. In a group of patients with advanced STS treated with radiotherapy alone, Tepper reported better local control in patients receiving 64 Gy or more.[9] Similar results were also reported by Slater *et al.* with better local control of tumour in patients receiving more that 65 Gy.[31] In a retrospective analysis by Tanabe and colleagues, a higher rate of local recurrence was seen in patients treated with pre-operative radiotherapy.[32] Levine *et al.* reported higher local recurrence rates in patients treated with a pre-operative dose of 25 Gy in 10 fractions with chemotherapy rates compared to those who received additional post-operative boost radiotherapy.[33] These data suggest that a dose response does exist in the radiotherapy treatment of STS. In our experience, patients treated with pre-operative radiotherapy to doses over 60 Gy had up to 80% response rates.[34]

The incidence of late morbidity is greater with use of a higher total dose of radiotherapy and higher dose per fraction. In the series by Slater, major complications were increased in patients treated to 70 Gy or more.[31] In a previous study performed at our centre, the degree of fibrosis was related to total dose of radiotherapy in patients treated for STS of the extremity.[35] Selch reported a significantly higher rate of toxicity in patients treated pre-operatively with high

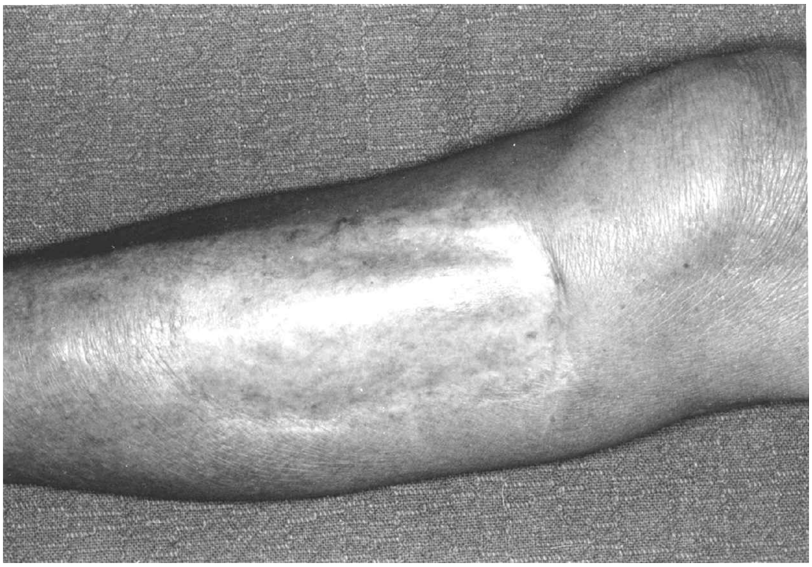

**Figure 4**. *Seven years after surgery and post-operative hyper-fractionated irradiation, the patient is free of disease. She enjoys good limb function despite mild joint stiffness.*

dose per fraction radiotherapy and concurrent chemotherapy.[36] Hence a total dose of 60–66 Gy in 2 Gy fractions is seldom exceeded in the treatment of these tumours, especially as the volumes treated are large and inevitably include a substantial amount of adjacent normal tissue.

Hyper-fractionated radiotherapy exploits the differences in fractionation response between tumour and normal tissue, by virtue of their different $\alpha/\beta$ ratios. Where the tumour has an $\alpha/\beta$ ratio exceeding that of late responding tissue, this technique could be used to deliver a higher total dose of radiation without an increase in late normal tissue damage.[18] Although studies on experimental tumours suggests a low $\alpha/\beta$ ratio in sarcomas[37] hyper-fractionation has not been extensively tested in the clinical setting.

There are only few published trials of hyper-fractionated radiotherapy in soft tissue sarcomas. Trials of combined chemotherapy and hyper-fractionated radiotherapy in paediatric Ewing's and rhabdomyosarcoma have shown promising results.[38,39] In a study by Mandell *et al.*, alternating chemotherapy and hyper-fractionated radiotherapy were used in gross residual or metastatic paediatric rhabdomyosarcoma. Patients were treated to a total dose of 54 Gy in 1.5 Gy fractions, treating twice a day with lesser acute toxicity and fewer treatment interruptions compared with concurrent chemotherapy and conventional radiotherapy.[38,40] Dunst *et al.* reported the use of hyper-fractionated radiotherapy in combination with chemotherapy in the treatment of paediatric Ewing's sarcoma.[39]

Willet *et al.* compared conventional radiotherapy to twice daily hyperfractionated radiotherapy (1.8–2 Gy fractions separated by 4 h) in STS of borderline resectability. Significantly better histological response was noted in the group treated using the hyper-fractionated regime. This suggested that hyperfractionated radiotherapy may be useful in the conservative surgical excision of STS of border-line resectability.[23] Hyper-fractionated radiotherapy has been used with radio-sensitizing agents in the treatment of advanced STS. In a group of 36 patients with unresectable STS at different sites treated with hyper-fractionated radiotherapy in 1.5 Gy twice daily fractions to a dose of 70–75 Gy in combination with Iododeoxyuridine, Goffman *et al.* reported 60% local control, with moderate toxicity.[41]

It is important to test the efficacy of hyper-fractionated radiotherapy prospectively with conventional fractionation. Accurate comparison of late toxicity between conventional and hyper-fractionated regimes is essential possibly to escalate the dose of radiation in the hyper-fractionated arm. Historical controls are of limited use in this regard and radiobiological predictions require clinical validation.

We propose to conduct a multi-arm trial, comparing hyper-fractionated with conventional radiotherapy in the post-operative treatment of STS. Patients with macroscopic residue following initial surgery are randomized for re-excision, followed by conventional or hyper-fractionated radiotherapy. Patients with complete excision or microscopic residual disease are randomized directly into the radiotherapy arms. This trial would help to assess the role of hyper-fractionated radiotherapy and surgical re-excision in local control of STS.

### Conclusion

It is feasible to deliver hyper-fractionated radiotherapy to a total dose of 72 Gy using twice daily 1.2 Gy fractions over 6 weeks in the treatment of STS of the extremity. The regime is well tolerated with comparable local control rates and late morbidity compared with standard irradiation. The advantage of this regime over conventional radiotherapy needs to be confirmed by a prospective randomized trial.

### References

1 Abbatucci JS, Boulier N, de Ranieri J, Mandard M, Tanguya, Busson A. Radiotherapy as an integrated part of the treatment of soft tissue sarcomas. *Radiother Oncol* 1984;2:115–21.

2 Lindberg RD, Martin RG, Romsdahl MM, Barkley HT. Conservative surgery and postoperative radiotherapy in 300 adults with soft-tissue sarcomas. *Cancer* 1981;47:2391–7.

3 Leibel SA, Tranbaugh RF, Wara WM, Beckstead JH, Bovill EG, Phillips TL. Soft tissue sarcomas of the extremities: survival patterns of failure with conservative surgery and post-operative irradiation compared to surgery alone. *Cancer* 1982;50:1056–83.

4 Rosenberg SA, Tepper J, Glatstein E, *et al.* The treatment of soft-tissue sarcomas of the extremities: prospective randomised evaluations of (1) limb sparing surgery plus radiation therapy compared with amputation and (2) the role of adjuvant chemotherapy. *Ann Surg* 1982;196:305–15.

5 Barkley HT, Martin RG, Romsdahl MM, Lindberg R, Zagars GK. Treatment of soft tissue sarcomas by preoperative irradiation and conservative surgical resection. *Int J Radiat Oncol Biol Phys* 1988;14:693–9.

6 Simon MA, Enneking WF. The management of soft tissue sarcomas of the extremities. *J Bone Joint Surg* 1976;58A:317–27.

7 Suit HD, Proppe KH, Mankin HG, Wood WC. Preoperative radiation therapy for sarcoma of soft tissue. *Cancer* 1981;47:2269–74.

8 Suit HD, Russel WO, Martin RG. Sarcoma of soft tissue: clinical and histopathologic parameters and response to treatment. *Cancer* 1975;35:1478–83.

9 Tepper EJ, Suit HD. Radiation therapy alone for sarcoma of soft tissue. *Cancer* 1985;56:475–9.

10 Shiu MH, Turnbull AD, Nori D, Hadju S, Hilaris B. Control of locally advanced extremity soft tissue sarcomas by function saving resection and brachytherapy *Cancer* 1984;53:1385–92.

11 Brennan MF, Hilaris B, Shiu MH, *et al.* Local recurrence in adult soft-tissue sarcoma—a randomised trial of brachytherapy. *Arch Surg* 1987;122:1289–93.

12 Morton DL, Eilber FR, Townsend CM Jr, Grant TT, Mirra J, Weisenburger TH. Limb salvage from a multidisciplinary treatment approach for skeletal and soft tissue sarcomas of the extremity. *Ann Surg* 1976;184:268–78.

13 Eilber FR, Mirra J, Grant TT, Weisenburger T, Morton DL. Is amputation necessary for sarcomas? A seven year experience with limb salvage. *Ann Surg* 1980;192:431–8.

14 Eilber FR, Giuliano AE, Huth J, Mirra J, Morton DL. Limb salvage for high grade soft tissue sarcomas of the extremitiy: experience at the University of California, Los Angeles. *Cancer Treat Symp* 1985;3:49–57.

15 Glaholm J, Harmer C. Soft tissue sarcoma: neutron versus photons for post-operative irradiation. *Br J Radiol* 1988;61:829–34.

16 Salinas R, Hussey DH, Fletcher GH, *et al.* Experience with fast neutron therapy for locally advanced sarcomas. *Int J Radiat Oncol Biol Phys* 1980;6:267–72.

17 Schmitt G, Rassow J, Schabel K, *et al.* Radiotherapy of soft tissue sarcomas with neutrons or a neutron boost. *Br J Radiol* 1984;57:247–50.

18 Withers RH, Peters LJ, Thames HD, Fletcher GH. Hyperfractionation. *Int J Radiat Oncol Biol Phys* 1982;8:1807–9.

19 Horiot JC, Le-Fur R, N'Guyen T, *et al.* Hyperfractionation versus conventional fractionation in oropharyngeal carcinoma: final analysis of a randomised trial of the EORTC cooperative group of radiotherapy. *Radiother Oncol* 1992;25:231–41.

20 Stuschke M, Thames HD. Hyperfractionated radiotherapy of human tumours: overview of the randomised clinical trials. *Int J Radiat Oncol Biol Phys* 1997;37:259–67.

21 Mandell LR, Ghavimi F, Exelby P, Fuks Z. Preliminary results of alternating combination chemotherapy (CT) and hyperfractionated radiotherapy (HART) in advanced rhabdomyosarcoma. *Int J Radiat Oncol Biol Phys* 1988;15:197–203.

22 Dunst J, Burgers JM, Hawlicek R, Trott KR, Jurgens H, Sauer R. Hyperfractionated radiotherapy with simultaneous chemotherapy in Ewing's sarcoma. *Strahlenther Onkol* 1988;164:30–32.

23 Willet CG, Schiller AL, Suit HD, Mankin HJ, Rosenberg A. The histologic response of soft tissue sarcoma to radiation therapy. *Cancer* 1987;60:1500–4.

24 Kinsella TJ, Rowland J, Glatstein E. Phase I/II study of continuous intravenous iododeoxyuridine (IdUrd) and twice daily irradiation for unresectable sarcomas (Abstr). *Am J Clin Oncol* 1986;9:113.

25 Robinson M, Cassoni A, Harmer C, Fisher C, Thomas J, Westbury G. High dose hyperfractionated radiotherapy in the treatment of extremity soft tissue sarcomas. *Radiother Oncol* 1991;22:118–26.

26 Robinson M, Barr L, Fisher C, *et al.* Treatment of extremity soft tissue sarcomas with surgery and radiotherapy. *Radiother Oncol* 1990;18:221–33.

27 Harmer C. Management of soft tissue sarcomas. In Selby P and Bailey C, eds. *Cancer and the Adolescent*, London: BMJ Publishing Group, 1996;69–89.

28 Jyothirmayi R, Sittampalam Y, Harmer C. Soft tissue sarcoma of the hand or foot: Conservative Surgery and Radiotherapy. *Sarcoma* 1999;3(1):17–24.

29 Harmer C and Bidmead M. Three-dimensional planning and conformal radiotherapy. In Verweij J, Pinedo HM, Suit HD, eds. *Soft Tissue Sarcomas: Present Achievements and Future Prospects*. Boston: Kluwer Academic Publishers, 1997;129–41.

30 Carabell SC, Goodman RL. Radiation therapy for soft tissue Sarcoma. *Semin Oncol* 1981;8:201–6.

31 Slater JD, Mc Neese MD, Peters LJ. Radiation therapy for unresectable soft tissue sarcomas. *Int J Radiat Oncol Biol Phys* 1986;12:1729–34.

32 Tanabe KK, Pollock RE, Ellis LM, Murphy A, Sherman N, Romsdahl MM. Influence of surgical margins on outcome in patients with preoperatively irradiated extremity soft tissue sarcomas. *Cancer* 1994;73:1652–9.

33 Levin EA, Trippon M, Gupta TKD. Preoperative multimodality treatment for soft tissue sarcomas. *Cancer* 1993;71:3460–71.

34 Robinson MH, Ball ABS, Schofield J, Fisher C, Harmer CL and Thomas JM. Pre-operative radiotherapy in the management if for extremity soft tissue sarcoma. *Clin Oncol* 1992;4:36–40.

35 Robinson MH, Spruce L, Eeles R, Fryatt I, Harmer CL, Thomas JM. Limb function following conservation treatment of adult soft tissue sarcoma. *Eur J Cancer* 1991;27:1567–74.

36 Selch MT, Kopald KH, Ferreiro GA, Mirra JM, Parker RG, Eilber FR. Limb-salvage therapy for soft-tissue sarcomas of the foot. *Int J Radiat Oncol Biol Phys* 1990;19:41–8.

37 Williams MV, Denekamp J and Fowler JF. A review of alpha/beta ratios for experimental tumours: implications for clinical studies of altered fractionation. *Int J Radiat Oncol Biol Phys* 1985;11:87–96.

38 Mandell LR, Ghavimi F, Exelby P, Fuks Z. Preliminary results of alternating combination chemotherapy and huperfractionated radiotherapy (HART) in advanced rhabdomyosarcoma (RMS). *Int J Radiat Oncol Biol Phys* 1988;15:198–203.

39 Dunst J, Sauer R, Burgers JM, *et al*. Radiotherapy in Ewing's sarcoma: current results of the German Society of Pediatric Oncology Studies. CESS 81 and CESS 86. *Klin Padiatr* 1988;200:261–6.

40 Jereb B, Ghavimi F, Exelby P, Zang E. Local control of embryonal rhabdomyosarcoma in children by radiation therapy when combined with chemotherapy. *Int J Radiat Oncol Biol Phys* 1980;6:827–33.

41 Goffman T, Tochner Z, Glatstein E. Primary treatment of large and massive adult sarcomas with iododeoxyuridine and aggressive hyperfractionated irradiation. *Cancer* 1991;67:572–6.
